# Significantly enhancement of sunlight photocatalytic performance of ZnO by doping with transition metal oxides

**DOI:** 10.1038/s41598-020-78568-9

**Published:** 2021-02-02

**Authors:** Alfonso E. Ramírez, Marly Montero-Muñoz, Lizbeth L. López, J. E. Ramos-Ibarra, Jose A. H. Coaquira, Benoît Heinrichs, Carlos A. Páez

**Affiliations:** 1 Grupo de Catalisis, Departmento de Química, Universidad del Cauca, Popayán - Cauca, Colombia; 2 Institute of Physics, University of Brasilia, Brasília, DF 70910-900 Brazil; 3 Grupo de Investigación en Electroquímica Y Medio Ambiente, Facultad de Ciencias Básicas, Universidad Santiago de Cali, Cali, Colombia; 4 Department of Chemical Engineering, Génie chimique—Nanomatériaux et Interfaces, Université de Liège, Liege, Belgium

**Keywords:** Materials science, Nanoscience and technology

## Abstract

In this study we report, the synthesis of ZnO and its doping with Transition Metal Oxides -TMO-, such as Cr_2_O_3_, MnO_2_, FeO, CoO, NiO, Cu_2_O and CuO. Various characterization techniques were employed to investigate the structural properties. The X-ray diffraction (XRD) data and Rietveld refinement confirmed the presence of TMO phases and that the ZnO structure was not affected by the doping with TMO which was corroborated using transmission Electron microscopy (TEM). Surface areas were low due to blockage of adsorption sites by particle aggregation. TMO doping concentration in the range of 3.7–5.1% was important to calculate the catalytic activity. The UV–Visible spectra showed the variation in the band gap of TMO/ZnO ranging from 3.45 to 2.46 eV. The surface catalyzed decomposition of H_2_O_2_ was used as the model reaction to examine the photocatalytic activity following the oxygen production and the systems were compared to bulk ZnO and commercial TiO_2_-degussa (Aeroxyde-P25). The results indicate that the introduction of TMO species increase significantly the photocatalytic activity. The sunlight photocatalytic performance in ZnO-doped was greater than bulk-ZnO and in the case of MnO_2_, CoO, Cu_2_O and CuO surpasses TiO_2_ (P25-Degussa). This report opens up a new pathway to the design of high-performance materials used in photocatalytic degradation under visible light irradiation.

## Introduction

The importance of raising global awareness about how light-based technologies promote sustainable development and provide solutions to global challenges has recognized by the United Nations^1^ In the environmental field, light can play a vital role, because its combination with semiconductor materials it provides a great power for photodegradation^2^. It is well known the use of semiconductors like TiO_2_^3^ and ZnO for this purpose^4^. Likewise, it is known that the wide band gap of these metal oxides limits their use in the visible range^5^. Additionally, rapid recombination of hole-electrons pairs is another limitation of ZnO^6^.

Therefore, the development of new generation nanophotocatalysts is a challenge for improving their photocatalytic activity in visible light. In the case of ZnO its application has been limited due to its large band gap^7^, which can decrease the photocatalytic properties^8^. One strategy to enhance their performance and its use with a light source, is doping with different elements and/or its compounds^9^. In case of ZnO, doping with the noble metals where Ag is most reported^10–13^, but we can find reports for Pd^14^, Pt^15^ and Au^16,17^. Other elements that have received special attention correspond to the lanthanide series^18^ specifically Ce^19,20^, Eu^21^, Gd^22,23^ and La^24^. Others metals of *d*-type that are also studied include Fe^25^, Ni^26,27^, Mn^28,29^, Co^30,31^ and Cu^32–34^.

The surface catalyzed decomposition of H_2_O_2_ has been reported as useful, simple and inexpensive way to evaluate the catalytic activity on solids under UV–visible light irradiation^35–37^. In this work, we have used the surface photodecomposition of H_2_O_2_ to show that doping ZnO with transition metal oxides -TMO-, enhance the photocatalytic powder of ZnO. In the case of doping with MnO_2_, Co_2_O_3_ and CuO, the resulting systems can become possible substitutes to most efficient commercial photocatalyst: Aeroxide TiO_2_/P25. This new information can be utilized to the future design of materials for the photocatalytic degradation under visible light irradiation.

## Results and discussion

### Characterization

**Figure 1:**
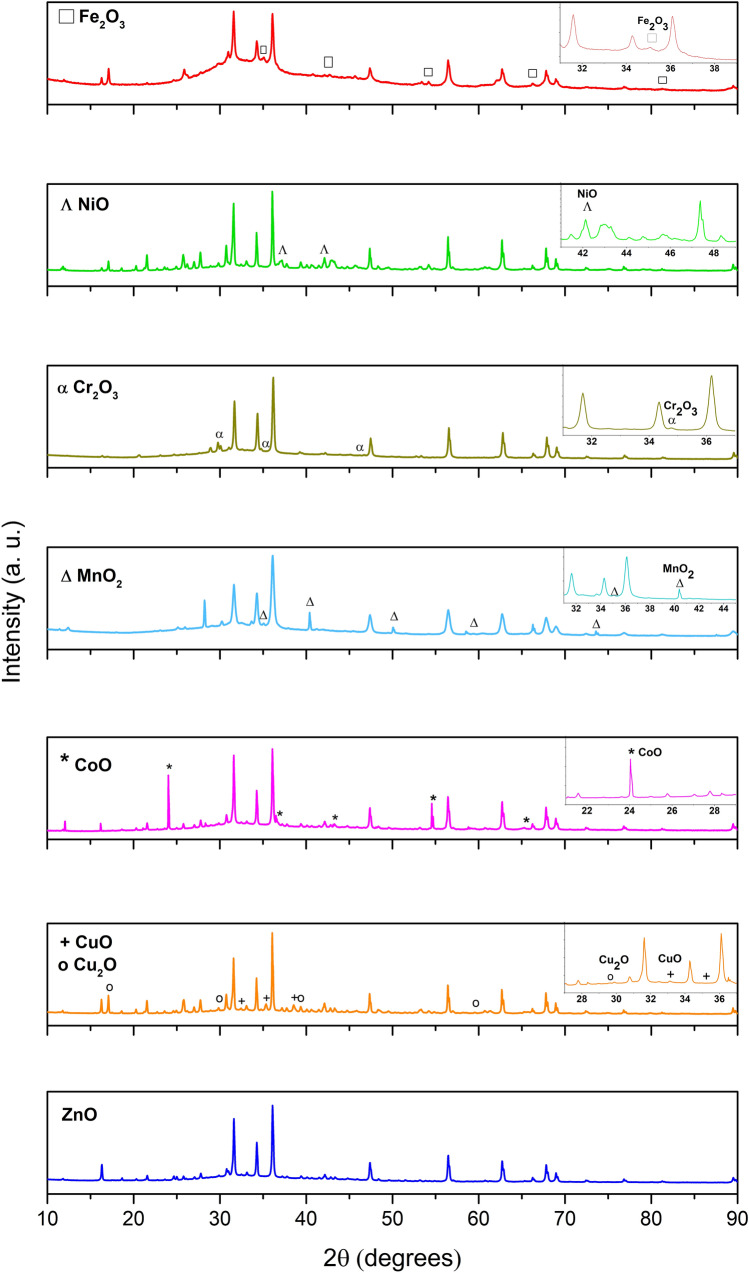
XRD patterns of transition metal oxides-doped ZnO nanoparticles. In the insets are shown reflections corresponding to the TMO dopant phase.

Zinc oxide phase corresponding to the wurtzite hexagonal structure (PDF 00–900-4179), with space group P63mc is the main phase as can be seen in Fig. 1. In addition to the ZnO main phase, reflections related to transition metal oxide dopant phase (see in the insets of Fig. 1) are determined. The following TMO phases were identified CuO (PDF 00–901-6057), Cu_2_O (PDF 00–900-7497), CoO (PDF 00–591-0031), Cr_2_O_3_ (PDF 00–900-7442), Fe_2_O_3_ (PDF 00–901-6457), NiO (PDF 00.432–0493) and MnO_2_ (PDF 00–151-4237).

In order to determine additional information, the XRD patterns were analyzed using the Rietveld refinement method. It was determined that the doping with TMO did not affect the wurtzite structure of ZnO, since all characteristic reflections of ZnO phase were also obtained in the XRD pattern of TMO/ZnO samples^38^. The lattice constants, unit cell volume and percentage of phases are listed in Table 1. In all of cases, the static structure factor of the wurtzite phase (c/a ~ 1.602) remains constant. It suggests that essentially no changes were determined in the lattice constants of the wurtzite phase, implying that the transition metal did not diffuse into the ZnO structure, in agreement with that reported in the literature^39^. Otherwise, the diffusion could lead to the substitution of Zn ions by the transition metal ions and it could lead to changes in the lattice constants of the wurtzite structure, which is not observed.

**Table 1:** Lattice parameters and percentage of phases obtained from the Rietveld refinement of the XRD patterns.

Catalyst	Wurtzite phase					TMO phase		
	a (Å)	c (Å)	c/a	V (Å^3^)	%	a (Å)	c (Å)	%
ZnO	3.2484	5.2046	1.602	47.56	100	−	−	0
CoO/ZnO	3.2498	5.2052	1.602	47.61	96	5.1842	3.0172	4
Cu_2_O–CuO/ZnO	3.2497	5.2060	1.602	47.61	76	4.6539	5.1083	16
						4.2521	4.2521	8
MnO_2_/ZnO	3.2514	5.2107	1.603	47.71	79	4.3886	2.8653	21
Cr_2_O_3_/ZnO	3.2485	5.2048	1.602	47.57	94	5.3524	5.3524	6
NiO/ZnO	3.2483	5.2037	1.602	47.55	89	4.1684	4.1684	11
Fe_2_O_3_/ZnO	3.2477	5.2028	1.602	47.59	82	5.4375	5.4375	18

**Figure 2:**
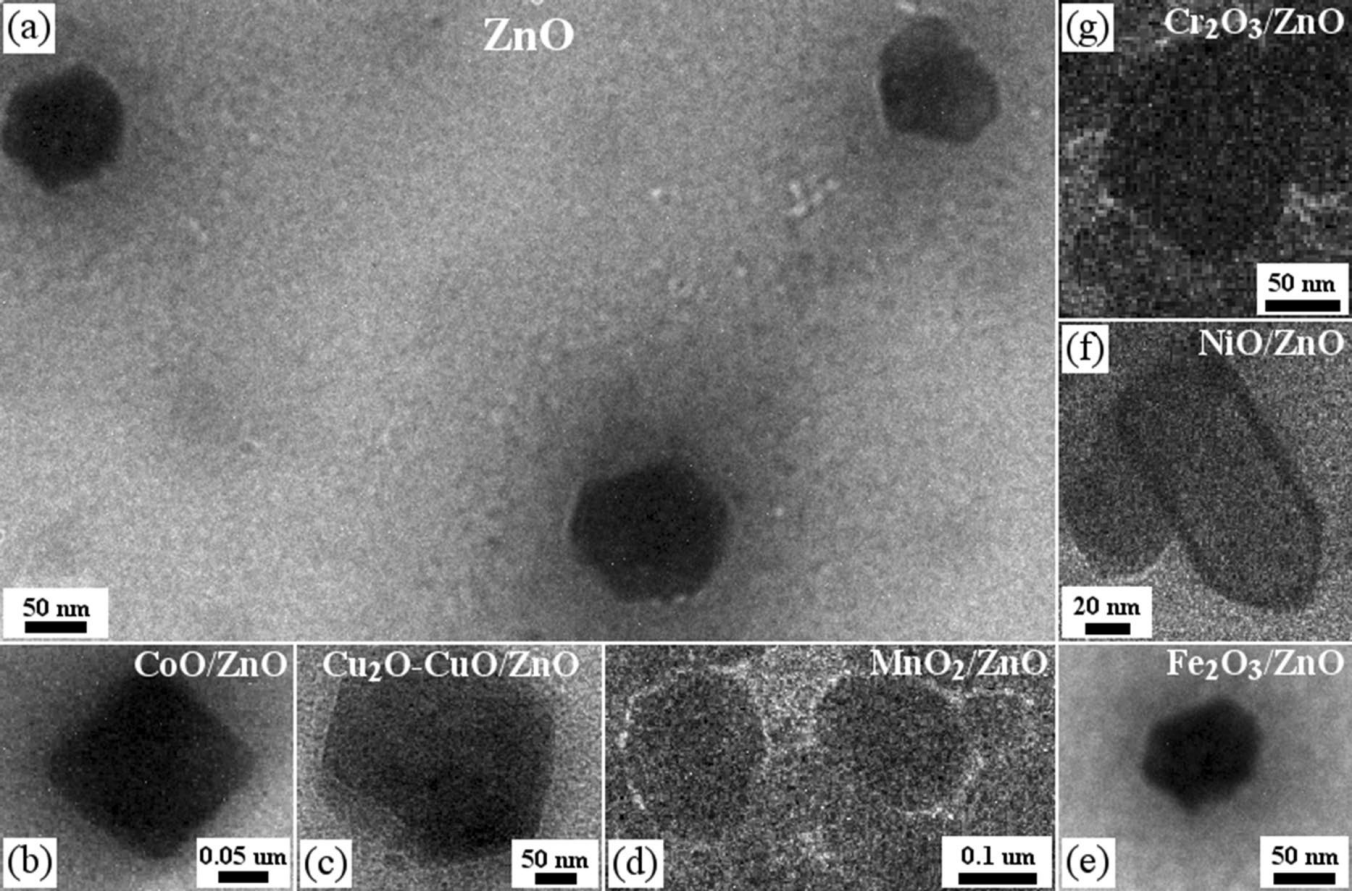
(**a**) ZnO nanoparticles obtained. (**b**) TMO-doped ZnO. (**c**) Cu_2_O-CuO-doped ZnO. (**d**) MnO_2_-doped ZnO. (**e**) Fe_2_O_3_-doped ZnO

TEM measurements were performed in order to get more information about the crystalline structure of the photocatalysts, mainly the particle size. Figure 2 shows the typical TEM images of the synthesized catalysts. Figure 2a shows the ZnO nanoparticles with a hexagonal structure and Fig. 2b presents ZnO powder modified with TMO.

As shown in Fig. 2a we can inferred the mean size of the ZnO nanoparticles, which is close to 47 ± 5 nm. In Fig. 2b is shown that the particles are in the nanoscale (approximately 50–60 nm in size) and reveals: (i) an increase in size, may be as a result of the TMO surface covering of ZnO nanoparticles^40^ and (ii) the conservation of hexagonal structure, indicating that the structure of ZnO is not notoriously affected by the TMO doping, in agreement with XRD data analysis.

The BET surface areas of our samples were determined and the values are listed in Table 2. ZnO exhibits a low surface area that reveal the effect of calcination as reported in the literature^41^*.* It is determined that the TMO-doped ZnO samples show smaller surface area in comparison to the surface area of pure ZnO. That surface area change was attributed to the particles aggregation and; thus, to the partial blockage of adsorption sites^42^.

**Table 2:** Main characteristics of catalysts used in the H_2_O_2_ photodecomposition.

Catalyst	%^a^ (w/w) M	S_BET_ (m^2^/g)	Band gap energy (eV)
ZnO	−	8.5	3.20
CoO/ZnO	4.3	2.5	2.46
Cu_2_O–CuO/ZnO	5.2	1.3	3.12
MnO_2_/ZnO	4.9	3.9	2.98
Cr_2_O_3_/ZnO	3.7	1.9	3.02
NiO/ZnO	5.0	2.5	3.15
Fe_2_O_3_/ZnO	5.1	4.8	2.92

^a^% (w/w) M represents the dopant content.

**Figure 3:**
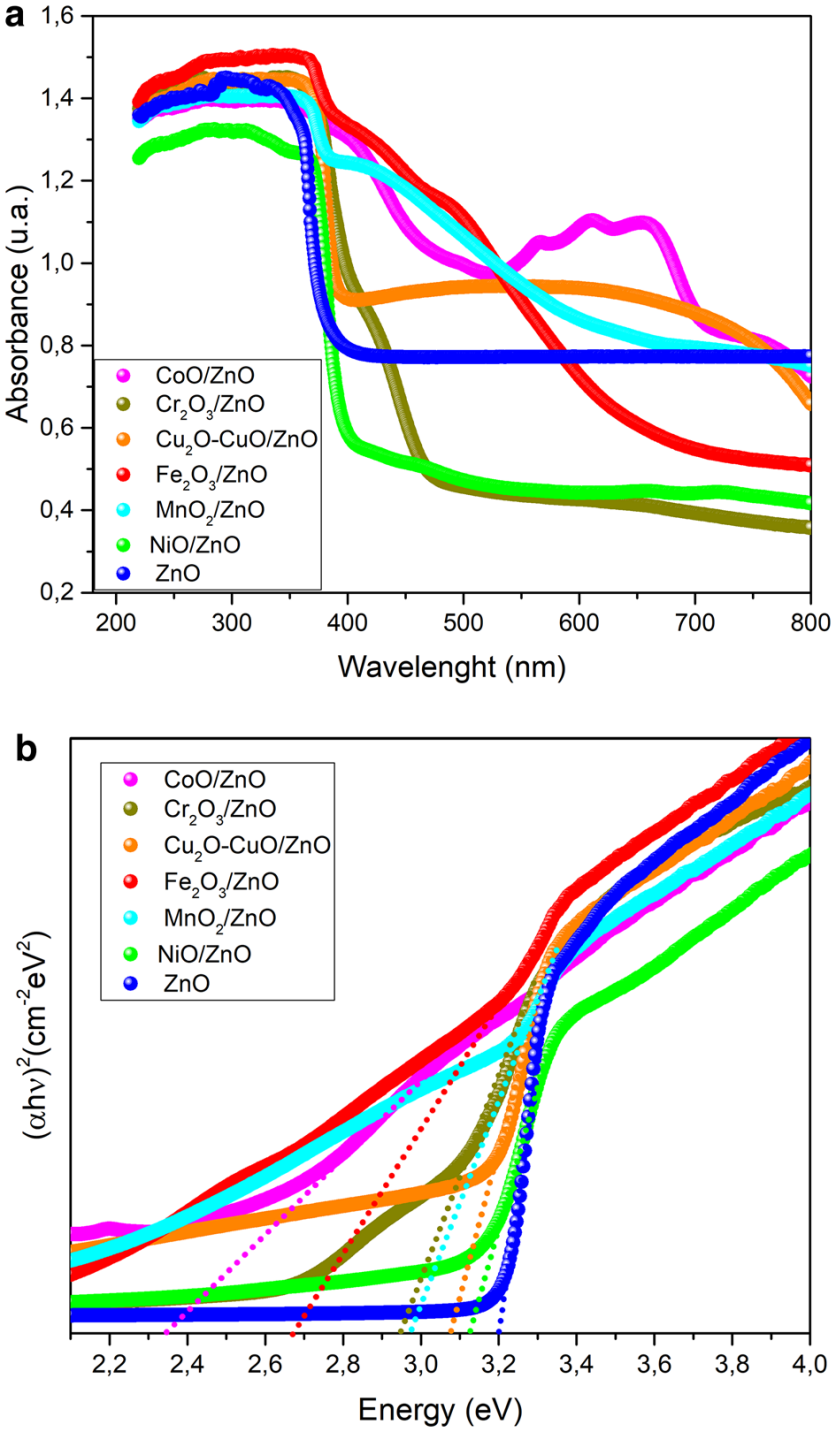
(**a**) UV–Vis spectra for photocatalysts. (**b**) Tauc´s plot for band *gap* determination of TMO-doped ZnO.

The optical characterization of materials allows the prediction of possible behavior of photocatalysts under illumination. Absorbance spectra, Fig. 3a, have been used to determine the optical band *gap* energy (E_g_). The values of E_g_ (Table 2) were determined using the Tauc’s plot method, Fig. 3b.

In all of cases, the band *gap* of TMO-doped ZnO are smaller in comparison to pure ZnO, being the smallest for the CoO/ZnO sample (2.46 eV). These results are in agreement with other works such as NiO/ZnO nanorods^43^ and CoO/ZnO nanofibers^44^. As a consequence of the coupling of MTO and ZnO in the heterojunction more electrons are freely transferred from M^n+^ of the TMO (with higher Fermi level) to ZnO (with lower level), promoting the separation of holes and electrons and, then, effective heterojunctions are formed^43^. Therefore, the band gap closing can facilitate stepping electrons from the valence band to the conduction band as that reported in the literature for CuO-ZnO nanocomposites^45^. That band gap closing leads to the photocatalytic activity improvement of the TMO-doped ZnO nanocomposites.

### Photocatalytic H_2_O_2_ decomposition

#### Control test

**Figure 4:**
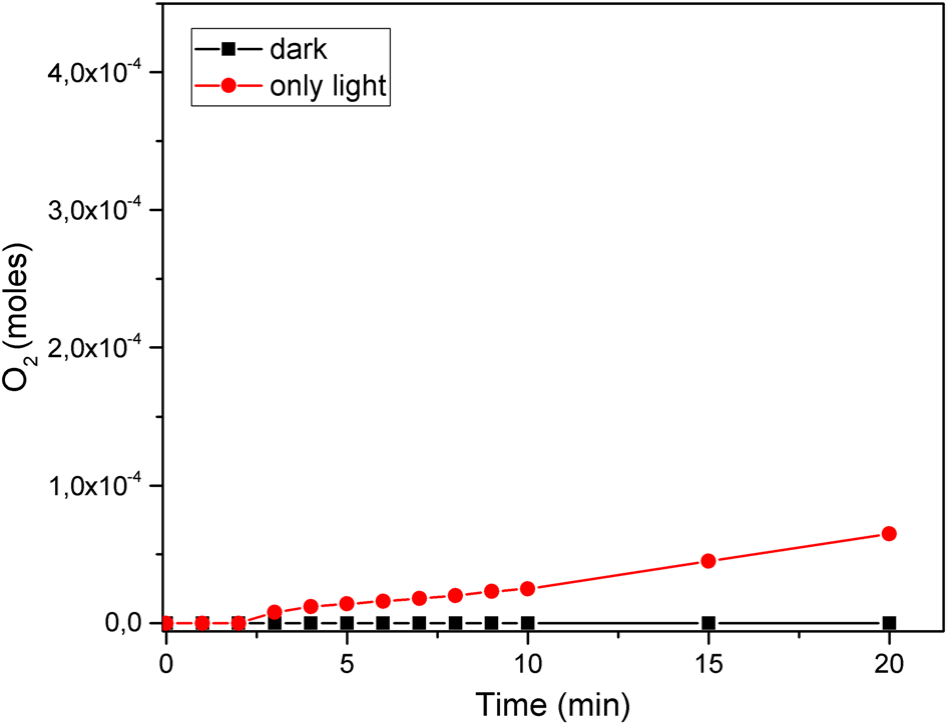
Control tests for the decomposition of H_2_O_2_.

The reaction in the dark condition as a function of time was followed. This control test confirmed that H_2_O_2_ is not decomposed in the dark condition. In the absence of catalyst but under visible light irradiation, 0.65 × 10^–4^ mol of O_2_ were produced and this control test allows us affirm that decomposition rates are related only to the effect of light on the TMO/ZnO systems (Fig. 4).

#### Photocatalytic systems

To understand the behavior of the materials in the reaction, we follow the O_2_ formation as a function of time under visible light irradiation.

The Table 3 shows the production of O_2_ form the decomposition of H_2_O_2_ during 20 min. As observed, there is a progressive increase in the O_2_ production. The conducting properties of the catalysts enhance the production of O_2_ and therefore the H_2_O_2_ decomposition.

**Table 3:** Production of O_2_ by H_2_O_2_ decomposition in presence of photocatalytic systems.

Catalysts	O_2_ production^a^ (mol × 10^–4^)
None	0.65
ZnO	1.00
CoO/ZnO	3.95
Cu_2_O–CuO/ZnO	3.99
MnO_2_/ZnO	3.66
Cr_2_O_3_/ZnO	2.16
NiO/ZnO	1.87
Fe_2_O_3_/ZnO	2.12
Aeroxide TiO_2_/P25	3.45

^a^During 20 min.

**Figure 5:**
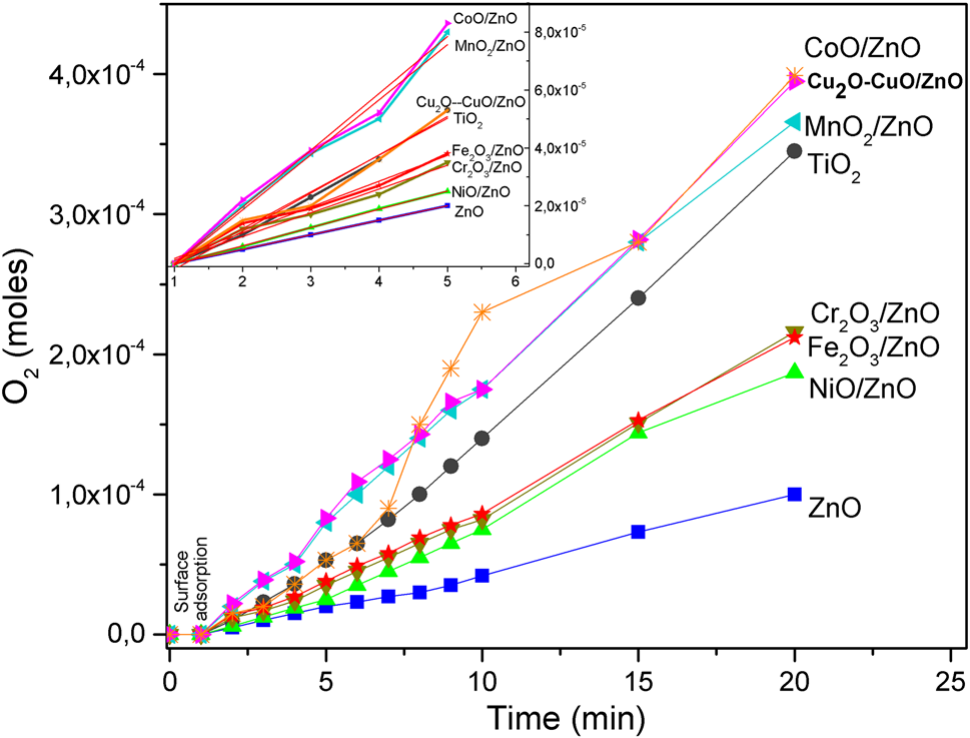
Kinetic study of the H_2_O_2_ decomposition carried out using the MTO-ZnO systems under light visible irradiation.

The Fig. 5 shows the kinetic study of the photocatalytic decomposition of H_2_O_2_ carried out using the TMO-ZnO systems. As observed, the O_2_ production obeys the first-order law, as previously reported in the literature^46^. The inset shows the initial rate (the slopes of the plots correspond to the rate constants). These rate constants are different and indicate distinct response of the systems, Table 4.

**Table 4:** Rate constants and catalytic activities for photocatalytic systems.

Catalysts	Rate constant (min^−1^ × 10^–5^)	Catalytic activity (O_2_ moles min^−1^ M^n+^ moles^−1^)
CoO/ZnO	1.96	5.4
Cu_2_O–CuO/ZnO	1.27	3.1
MnO_2_/ZnO	1.90	4.2
Cr_2_O_3_/ZnO	0.82	2.3
NiO/ZnO	0.63	1.5
Fe_2_O_3_/ZnO	0.89	1.9

The irradiation without catalysts just promotes a weak H_2_O_2_ decomposition and leads to the O_2_ production 0.65 × 10^–4^ mol, while the use of ZnO increases the O_2_ production up to 1.5 times (Table 3) demonstrating the catalytic power of ZnO. Nevertheless, the doping of ZnO with MTO significantly enhance the photocatalytic power of ZnO, which suggests that the surface charge transfer process should increase, meanwhile, the electron—hole recombination rate should decrease^42^. In fact, the O_2_ production (Table 3) can go up to 3.95 × 10^–4^ mol in the presence of CoO/ZnO, which seems to be the system with the highest activity. Additionally, it was determined that the Aeroxide TiO_2_-P25 power can be exceed by the ZnO doping with transition metal oxides, specially Cu_2_O, CuO, MnO_2_ and CoO.

Table 4 shows the reaction rate and the catalytic activity values. These parameters corroborate that the best dopant oxides are those previously mentioned. These results have been correlated with the redox potential and the amount of loaded metal ion^47^. Mn^+4^, Cu^+2^, Co^+2^, Cu^+1^ and Fe^+3^ have positive values and the low rate observed with Cr^+3^ and Ni^+2^ is in agreement with their negative redox potentials^48^.

The introduction of TMO in ZnO improves its absorption in the visible region, Fig. 3a This leads to reduce the extent of undesired recombination of charge carrier resulting in a better activity^49^ and a decrease of the value of band gap, which leads to higher efficiency of photocatalysts due to a better overlap with the light source spectrum^50^.

The H_2_O_2_ decomposition under visible light irradiation by different semiconductors SC as TiO_2_^51^ or MnO_2_^35^ has been studied and the mechanism is based on the oxidation–reduction properties of SC. For ZnO can be understood as follows: the irradiation of ZnO leads to excited state that can be expressed as ZnO (e^−^, h^+^) (reaction R.1). The electron in the conduction band -CB- is available for transference (reaction R.2) while photoinduced valence band holes is open for donation (reaction R.3).

R.1 $${\text{ZnO }}\xrightarrow{{hv}}{\text{ ZnO }}\left( {{\text{e}}^{ - } ,{\text{h}}^{ + } } \right)$$

R.2 $${\text{h}}^{ + } + {\text{ H}}_{2} {\text{O}}_{2} \to {\text{H}}^{ + } + {\text{ HO}}_{2}^{ .}$$

R.3 $${\text{e}}^{ - } + {\text{ H}}_{2} {\text{O}}_{2} \to {\text{HO }}^{.} + {\text{ OH}}^{ - }$$

R.4 $${\text{H}}_{2} {\text{O}}_{2} \to 1/2{\text{O}}_{2} + {\text{ H}}_{2} {\text{O}}$$

A direct interfacial charge transfer mechanism is proposed for the visible light activity in M^n+^-modified samples^52^. Firstly, the adsorption of H_2_O_2_ by the TMO/ZnO system releases H^+^ ions (reaction R.5). The electrons transferred to the CB of TMO from the valence band -VB- of ZnO lead to the metal reduction (reaction R.6). The catalyst regeneration step involves the HO^.^ reduction to HO^-^ (reaction R.8).

R.5 $${\text{M}}^{{{\text{n}} + }} /{\text{ZnO}} + {\text{ H}}_{2} {\text{O}}_{2} \to {\text{ M}}^{{{\text{n}} + }} { }/{\text{ ZnO }}({\text{HO}}_{2} )^{ - } + {\text{ H}}^{ + }$$

R.6 $${\text{ M}}^{{{\text{n}} + }} { }/{\text{ ZnO }}({\text{HO}}_{2} )^{ - } + {\text{ H}}^{ + } { }\xrightarrow{{{\text{hv}}}} {\text{ M}}^{{\left( {{\text{n}} - 1} \right) + }} /{\text{ZnO}} + {\text{ HO}}_{2}^{{{ }.}}$$

R.7 $${\text{HO}}_{2}^{{{ }.}} + {\text{ H}}_{2} {\text{O}}_{2} \to {\text{HO }}^{.} + {\text{ O}}_{2} + {\text{ H}}_{2} {\text{O}}$$

R.8 $${\text{M}}^{{\left( {{\text{n}} - 1} \right) + }} /{\text{ZnO }} + {\text{ HO }}^{.} \to {\text{M}}^{{{\text{n}} + }} /{\text{ZnO}} + {\text{ HO}}^{ - }$$

Therefore, in the two pathways, the free radicals HO_2_^.^ (R.2 and R.6) or HO^.^ (R.3) induce the chain reactions sequence to produce the final products, H_2_O and O_2_ (reaction R.4 and R.7).

## Methods

### Preparation of ZnO and MTO-doped ZnO

ZnO and MTO-doped ZnO were prepared by the sol–gel method using a precursor alkaline solution composed of zinc acetate dihydrate dissolved in methanol, as described in a literature^53^. In all cases, the dopant source of MT was nitrate except in the case of Mn, which was chloride. In a representative preparation, ZnO and MTO-doped ZnO were synthesized by the slow hydrolysis of zinc acetate using KOH as precursor. Zinc acetate dehydrate and a dopant were first dissolved in methanol and mixed together with a KOH solution (0.4 M) for obtain a clear and homogeneous solution. The solution was stirred at 60 °C for 2 h. Finally, the gel washed, dried and powdered before calcinations at 450 °C for eight hours in a muffle furnace. In Table 2 is shown the doped metal content determined from XRF measurements.

### Characterization of ZnO and MTO-doped ZnO

X-ray diffraction analysis was performed using a RIGAKU Ultima IV diffractometer, with a Cu-K_α_ as radiation source and Ni-filtered with CBO monochromator. Operating voltage was 45 kV with beam current of 15 mA. The measurements were performed at step widths of 0.05 and the scan rate was maintained at 2° min^−1^. The XRD of the samples were performed in the 2θ range of 20–70^o^ as in^54^. Additionally, the XRD data were analyzed using the Rietveld refinement method via the FullProf program. QUALX2.0 software was used for phase identification in the qualitative analysis from powder diffraction data.

Transmission electron microscopy (TEM) images were obtained by using a microscope (JOEL, model 1011) to determine the morphology, the mean particles size and the size distribution as in^54^.

The content of metal was estimated by Energy Dispersive X-ray technique using a EDX-720 Shimadzu Fluorescence Spectrometer (XRF). The solids were prepared as loose powder. The analysis was made using a Rh X-ray tube for 200 s under vacuum.

The UV–Vis spectra of samples were recorded in the range of 220–1000 nm using UV-2600 Shimadzu spectrophotometer.

For nitrogen adsorption–desorption, the measures were made at − 196 °C with a Fisons Sorptomatic 1990, after outgassing at 10^−3^ Pa for 24 h at ambient temperature.

### ***Photocatalytic degradation of H***_***2***_***O***_***2***_

Degradation of H_2_O_2_ under visible light simulated conditions, with an halogen lamp (300 W, 220 V, λ ≥ 400 nm), was used to evaluate the photocatalytic activity of ZnO and MTO-doped ZnO following the report made by Paéz et al*.*^35^. The initial pH was kept between 4.6–5.1. 5 mg of solid was suspended in 50 mL of deionized water in the reactor and ultra-sounded for 30 min; when the temperature was 20 °C, 10 mL of H_2_O_2_ solution were injected into the solid suspension and the lamp was turned on under visible light radiation. The production (in mol) of oxygen was calculated by the change in H_2_O_2_ concentration during photocatalytic run and has been determined from Eq. (1).

1 $$C = Co - 2 \times \frac{{PV_{g} }}{{RTV_{L} }}$$

where C is the concentration of H_2_O_2_ at time t (mol L^−1^), C_0_ the initial concentration of H_2_O_2_ (6.5 mol L^−1^), P the atmospheric pressure (≅ 101.3 kPa), R is the gas constant (8.314 L kPa mol^−1^ K^−1^), V_L_ the total volume of solution (0.015 L), T is the room temperature and V_g_ corresponds to the integrated volume of gas liberated until time t (L) at atmospheric pressure measured by the devices.

## Conclusion

TMO/ZnO nanocomposites were successfully synthesized and tested their photocatalytic activity for H_2_O_2_. The morphological and structural results confirmed that the TMO doping did not provoke the ions substitution in the ZnO lattices and that the ZnO structure is not affected. Optical measurements showed the ZnO band gap decrease with the doping. It is found that the doping reduces the electron–hole recombination rate, which improves the absorption in the visible region and leads to a significantly enhancement of sunlight photocatalytic performance of ZnO. The surface-mediated decomposition of H_2_O_2_ and the consequent production of O_2_ were used to measure the photocatalytic power of the TMO/ZnO nanocomposites. The resultant reaction rate values are explained based on the combination of redox potential of metal of the TMO and a better absorption of visible light due to the presence of TMO in the TMO/ZnO system. Our findings indicate that Cu_2_O/ZnO, CuO/ZnO, MnO_2_/ZnO and CoO/ZnO systems overcomes the photocatalytic activity of most popular commercial photocatalyst: Aeroxide P25. Therefore, our results indicate that TMO/ZnO systems can substitute the current commercial photocatalysts.
